# Alcohol Consumption Reduction Among a Web-Based Supportive Community Using the Hello Sunday Morning Blog Platform: Observational Study

**DOI:** 10.2196/jmir.9605

**Published:** 2018-05-17

**Authors:** Jessica Jane Louise Kirkman, Briony Leo, Jamie Christopher Moore

**Affiliations:** ^1^ Hello Sunday Morning Surry Hills Australia

**Keywords:** alcohol drinking, internet, Web-based brief alcohol intervention, moderate drinking, alcohol use, alcohol abuse, binge drinking, internet intervention, relapse prevention, drinking behavior, alcoholic intoxication, social network, blogging, blog search, internet media, platforms, community, engagement

## Abstract

**Background:**

Alcohol misuse is a major social and public health issue in Australia, with an estimated cost to the community of Aus $30 billion per annum. Until recently, a major barrier in addressing this significant public health issue is the fact that the majority of individuals with alcohol use disorders and alcohol misuse are not receiving treatment.

**Objective:**

This study aimed to assess whether alcohol consumption changes are associated with participation in Hello Sunday Morning’s blog platform, an online forum discussing experiences in abstaining from alcohol.

**Methods:**

The study reports on Hello Sunday Morning participants who signed up for a 3-month period of abstinence from November 2009 to November 2016. The sample comprised 1917 participants (female: 1227/1917, 64.01%; male: 690/1917, 35.99%). Main outcome measures were Alcohol Use Disorders Identification Test (AUDIT) scores, mood, program engagement metrics, and slip-ups.

**Results:**

Individuals who reported hazardous (preprogram AUDIT mean 11.92, SD 2.25) and harmful consumption levels (preprogram AUDIT mean 17.52, SD 1.08) and who engaged in the Hello Sunday Morning program reported a significant decrease in alcohol consumption, moving to lower risk consumption levels (hazardous, mean 7.59, SD 5.70 and harmful, mean 10.38, SD 7.43), 4 months following program commencement (*P*<.001). Those who reported high-risk or dependent consumption levels experienced the biggest reduction (preprogram mean 25.38, SD 4.20), moving to risky consumption (mean 15.83, SD 11.11), 4 months following program commencement (*P*<.001). These reductions in risk were maintained by participants in each group, 7 months following program commencement. Furthermore, those who engaged in the program more (as defined by more sign-ins, blogs posted, check-ins completed, and engagement with the community through likes and following) had lower alcohol consumption. Finally, those who experienced more slip-ups had lower alcohol consumption.

**Conclusions:**

Participation in an online forum can support long-term behavior change in individuals wishing to change their drinking behavior. Importantly, reductions in AUDIT scores appeared larger for those drinking at high-risk and hazardous levels before program commencement. This has promising implications for future models of alcohol reduction treatment, as online forums are an anonymous, accessible, and cost-effective alternative or adjunct to treatment-as-usual. Further research is needed into the specific mechanisms of change within a Web-based supportive community, as well as the role of specific mood states in predicting risky drinking behavior.

## Introduction

### Background

Alcohol misuse is a major social and public health issue in Australia, with an estimated cost to the community of Aus $30 billion per annum [1,2]. In Australia, alcohol consumption is estimated to cause 3.2% of the total burden of disease, contributing approximately 188,000 disability adjusted life years, causing 5550 deaths per annum [1,2]. In high-income and middle-income countries, the costs associated with alcohol misuse amount to more than 1% of the gross national product, represented largely in social harm and health costs [3]. Government policies targeted at reducing alcohol consumption have focused on education about the risks and harms of alcohol consumption and reducing the accessibility and affordability of alcohol.

Although alcohol misuse and alcohol use disorder (AUD) can be seen as distinct from each other, it can be argued that they are on the same trajectory. Most theories of alcohol dependency describe high-risk drinkers experiencing nervous system adaptations from repeated alcohol exposure—a process which underlies the development of an alcohol addiction [4,5].

Until recently, a major barrier in addressing this significant public health issue is the fact that the majority of individuals with both high-risk drinking and AUDs are not receiving treatment [6]. For this population, a significant treatment gap exists whereby a *hidden* population of individuals with problematic alcohol use is dissuaded from seeking help due to a lack of accessibility of services, stigmatization, low motivation, or cost of treatment [7]. Furthermore, substantial evidence exists to show that those with less severe alcohol abuse are not offered interventions, and those who are gainfully employed are even less likely to receive any type of treatment [8]. What is notable though is that most alcohol-related harm arises from those who consume in an “at-risk” manner rather than those with an AUD [9].

This *hidden* population of individuals with problematic alcohol use is a prime candidate for internet-based interventions, as a first step toward seeking help [10]. It is this population who are difficult to engage in traditional treatment services but from whom internet-based interventions are ideal due to their low cost and relative convenience. Internet-based interventions have promising applications in the treatments of addiction, with advantages including anonymity, convenience, accessibility, cost-effectiveness, and privacy [11,12]. Internet therapies can also target clients in different stages of change [13], allowing potential clients to investigate treatment options without the shame and guilt associated with face-to-face interactions. Additionally, these therapies have the potential to be more consistent than face-to-face therapy delivery, with structured programs that are delivered with clinical fidelity [14].

Internet-based interventions can also provide the context for screening and brief intervention (SBI) for those drinking to “at-risk” levels. SBI has a growing evidence base for its effectiveness [15-18]; however, there is an absence of evidence for those experiencing more severe alcohol use problems [15,17,19]. Reviews of previous studies of electronic SBIs have also found inconsistent evidence as to their effectiveness with high-risk drinkers, with an acknowledgment that, although Web-based interventions appear to be well received, further controlled trials examining engagement and efficacy are needed within this population [20].

It is important to acknowledge that, in this emerging field, there are many terminologies to describe interventions delivered over the internet. Common terms include e-mental health, Web-based-therapy, mHealth, cybertherapy, and telemental health. eHealth describes the range of modalities that deliver therapeutic services—such as telephone, internet, email, and videoconferencing [21]. This differs from telerehab, which is rehabilitation-focused rather than a psychological or psychosocial intervention. For ease of reference, in this paper, the term eHealth will be used to describe services delivered online.

In terms of treatment, the existing evidence for eHealth shows promising results across multiple populations and demographics [22-26]. Again, the populations targeted in most of the research have been for problematic drinking rather than those with diagnosed AUDs, making it difficult to ascertain whether these interventions are useful for those with a serious drinking problem. More recent research that has focused on individuals with a specifically diagnosed AUD has found evidence for the efficacy of a stand-alone mobile phone alcohol reduction app, as well as internet-based interventions with bibliotherapy [27]. For both of these conditions, greater utilization of the system provided greater results over the course of the study, and participants had control over how quickly they progressed through their treatment—and were able to access the material when needed, in contrast to more traditional treatment services. The research into eHealth explores benefits such as cost-effectiveness, flexibility of design, replicability, interactivity, and ease of use, as well as the question of whether these interventions are as effective as face-to-face programs, and as adjuncts to treatment in primary care settings [28].

In terms of less structured or formalized Web-based interventions, the internet also provides individuals the opportunity to connect with others and participate in activities such as blogging. Blogging has gained popularity over the past 10 years, with most blogs fitting the description of regular, date stamped articles that represent a timeline, and are used by the author for personally oriented communication [29]. They differ from traditional print and digital media in terms of their flexibility, interactivity, informal structure, and engagement with readers, with users having the opportunity to express their opinions on posts and in a public forum. Research into internet users has identified convenience, a sense of community, and information seeking as the major reasons for visiting blogs [30]. The impacts of blogging can also be behaviorally empowering and tie in with intrinsic motivations [31], as well as providing a cathartic space to process difficult emotions [32].

Research has also established that a person’s social network can either increase or decrease their alcohol use [33]. Today, that includes an individual’s online social network. These social networks can allow a user to post a blog with text and images or engage in group discussions [34]. Although the impacts of social networks and blogging on behavior change are inconsistent in relation to diet and exercise [35], their impact on reducing alcohol consumption has not been determined [34,35].

### Hello Sunday Morning

**Figure 1:**
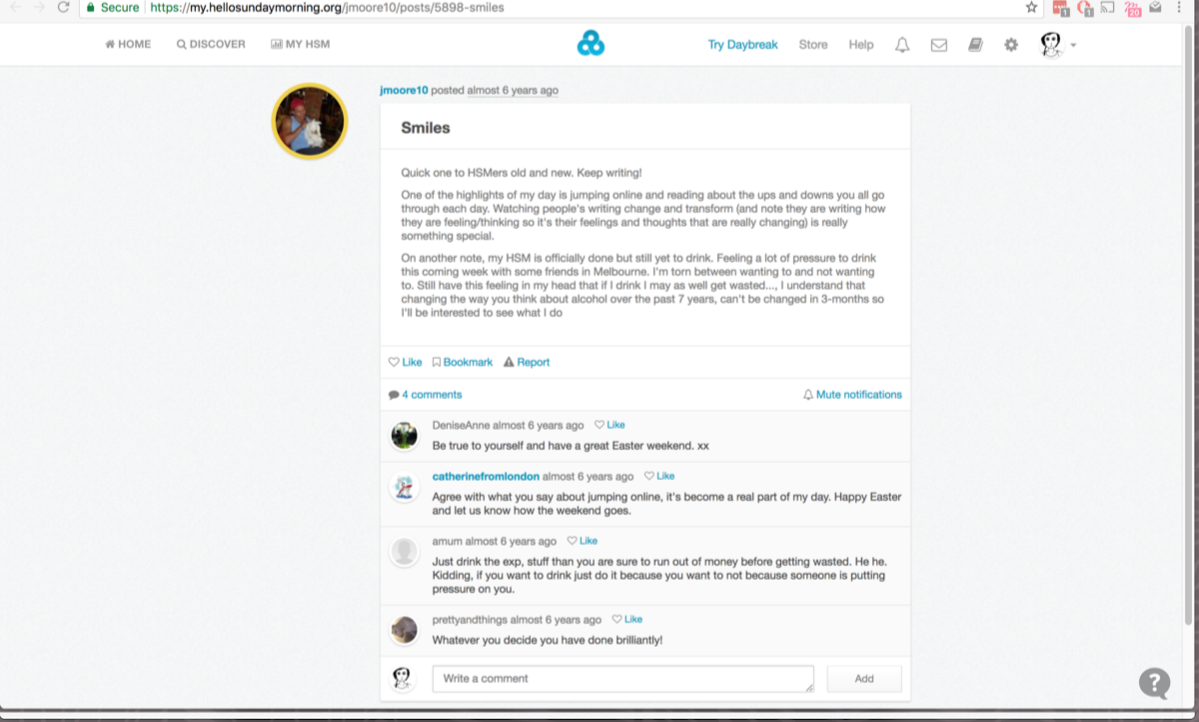
Sample blog post (Hello Sunday Morning, 2018).

**Figure 2:**
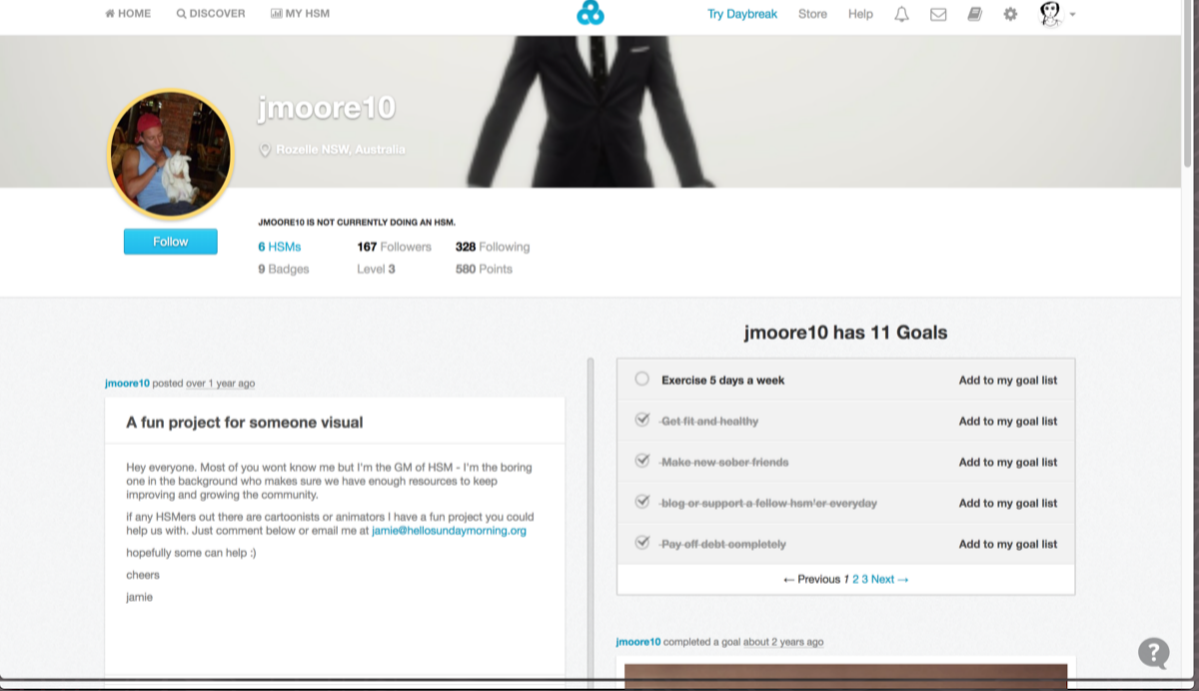
Sample profile of Hello Sunday Morning (HSM) member (Hello Sunday Morning, 2018).

Hello Sunday Morning (HSM) is an Australian social media health promotion “movement” that asks participants to publicly set a personal goal to stop drinking or reduce their consumption, for a set period of time, and to record their reflections and progress on blogs and social networks (see Figures 1 and 2). The action of setting a goal both motivates members and holds them accountable, while also creating a message about healthy relationships with alcohol within their peer group. Created in 2010, HSM has developed a Web-based platform that combines blogging, social media, and gamification (structured games that facilitate participation and engagement with the Web-based community). HSM is unique in that the program participants produce all of the content based on their individual experiences, using the vernacular of their peer group.

HSM has effectively built a strong culture that governs the norms and values of the community. Carah et al [36] suggested that HSM—like fitness apps, quit smoking initiatives, and mindfulness programs—is worth studying to understand how social media health promotion and treatment can overlap.

Preliminary research has been conducted on the HSM platform, exploring blog content and qualitative reports of change. First, text analytics of the blog posts on the platform show that participants typically begin with descriptions of their drinking practices and change over time to reflect their efforts at change, and their aspirations [37]. Early evaluations have also revealed that 84% of Australian HSM users reported completing the program time without a “slip-up” (defined as a drink before finishing the program), reduced their alcohol use, reported improved mental health, and experienced a change in their perceptions of alcohol over time [38]. Specifically, participants reported increased understanding of the negative effects of alcohol, a decrease in the desire to drink alcohol for fun, and a decrease in the likelihood to drink alcohol to relieve tension. Further evaluations have demonstrated members shift from being self-focused to reflecting on the role of alcohol in society and developing a desire to support others [38]. A study of high-risk Victorian HSM participants also found that the majority of participants reported low-risk drinking at 1 month and reported improved physical health, feeling positive about themselves, greater productivity, engagement in new activities, improved mental health, new or improved relationships, and financial savings [39].

More recent research on the HSM platform and its participants has revealed that 64% of participants were under the age of 40 years, were more likely to be female (which is the opposite of the Australian alcohol treatment population), and are riskier drinkers than other treatment-seeking populations [36]. These participants most commonly selected “fitness” and “mind and body” as their goal, whereas “sobriety” was the third most significant goal. Those drinking at high-risk levels were twice as likely to elect “alcohol”-related goals, compared with those drinking at lower risk levels. Carah et al [36] concluded that through qualitative analysis, they believe heavier drinking participants may use HSM as a treatment program. As such, it was suggested that further research is required to examine whether participants self-reported alcohol consumption changes after participation [36].

### This Study

This study, therefore, aimed to address outstanding questions on whether alcohol consumption changes are associated with participation on the HSM platform and explore the reasons why this may be the case or what factors are driving the change. The primary outcome will be change in Alcohol Use Disorders Identification Test (AUDIT) scores. The AUDIT was developed by the World Health Organization and has been validated for use in Australia [40]. The AUDIT has been used as an outcome measure in previous evaluations of the HSM platform [38,39] as well as other clinical populations [41-44]. Although the gold standard for evaluating service efficacy is a randomized controlled trial design, such a design would be impractical when offering this service for the first time to the previously described *hidden* population who are not traditional treatment seekers. To allow for a preliminary trial of the technology, the Web-based program was openly offered to all eligible participants. This study is the first in a series examining the service, its reach, and its therapeutic components, including a randomized controlled trial currently under way.

In this study, the following was hypothesized: (1) that participants’ AUDIT score will significantly decrease from preprogram to 1-month follow-up (4 months after program commencement), and this will be maintained at 3-month follow-up (7 months after program commencement), and further, that this change will occur in participants across all 3 AUDIT risk zones; (2) that 1- and 3-month AUDIT change scores will be negatively correlated with program engagement metrics including the number of sign-ins, posts, comments received, comments given, followers, and people following; (3) that 1- and 3-month AUDIT change scores will be negatively correlated with mood and positively correlated with slip-ups.

## Methods

### Participants

This study reports data on HSM participants who have signed up for a 3-month period of abstinence from November 2009 to November 2016. Although some participants complete multiple HSM periods of abstinence, data reported in this study reflect participants’ first engagement with the program. Participants classified as low-risk consumption (as measured by Zone 1 on the AUDIT) were excluded from the sample used in analyses, as they were not identified as having unhealthy drinking levels. The resulting sample consisted of program participants who signed up to complete one 3-month HSM experience, screened as Zones II, III, or IV on the AUDIT at program commencement, signed in at least once, and completed at least 1 follow-up survey (ie, 1- or 3-month).

### Materials

#### Alcohol Use Disorders Identification Test

The AUDIT is a 10-item measure assessing alcohol consumption [40]. Participants are required to respond to items (eg, “How often do you have 6 or more drinks on one occasion?”) on a 5-point Likert scale, with each question receiving a score with a range of 0-4 points, with a potential maximum total score of 40 points. In Australian usage of the AUDIT, a score between 0 and 7 indicates low-risk consumption, a score between 8 and 15 indicates risky or hazardous consumption, a score between 16 and 19 indicates harmful consumption, and a score of more than 20 indicates high-risk or dependent consumption [45].

#### Mood

Mood was measured by a single item (“How did you feel this week?”) asked at each weekly check-in, with members responding on a 4-point visual scale of faces ranging from very happy to very sad.

#### Slip-Ups

Slip-ups were measured by a single item (“How did you go this week?”) asked at each weekly check-in, with members responding with either “I’m still going strong,” indicating they did not drink, or “I slipped up,” indicating they did drink.

#### Program Engagement

Program engagement is measured by a number of factors that were automatically logged within the website. This includes a count of the total number of sign-ins, posts made on the platform, comments received, comments given, followers, and people following the member. The ability to capture these metrics was intentionally created by the website developers, rather than relying on other metrics such as Google analytics.

### Procedure

When participants register on the HSM Web platform, they accept the terms and conditions which state that information about their participation and alcohol use is used in research evaluations. Participants, therefore, provided informed consent for their deidentified data to be used for research purposes. Three months following the completion of their 3-month abstinence period, they are sent an email prompting them to check in to see how their relationship with alcohol has changed since signing up. They are offered a 25% discount on any HSM store purchase if they complete the follow-up survey within 48 hours.

### Data Analysis

Results are presented for 2 different sets of data analyses. Data were cleaned and checked for appropriate assumptions of normality, sphericity, and outliers in line with the analyses conducted. First, a set of repeated measures analysis of variance (ANOVA) was conducted to explore reductions in AUDIT score following program completion. Then, separate bivariate Pearson correlations were conducted to determine associations with 1- and 3-month follow-up AUDIT change scores, with various program engagement metrics, and with mood and slip-ups. AUDIT change scores were computed by subtracting program commencement AUDIT score from 1- and 3-month follow-up AUDIT score, with the resulting variable indicating negative scores for AUDIT reduction and positive scores for AUDIT increase. All ANOVAs were conducted, with an overall alpha of .01 to provide a more conservative control of type-I error, with all correlational analyses conducted at an alpha of .05 as they were observational in nature.

## Results

### Demographic Information

Participants were 1917 adults with an average age of 46 years (SD 11.71). Overall, 64.00% (1227/1917) of participants were female and 35.02% (690/1917) were male, with the highest proportion reporting they reside in Australia (709/1917, 36.99%), participants followed by the United States (498/1917, 25.98%), the United Kingdom (249/1917, 12.99%), and Canada (153/1917, 7.98%). In total, 25.98% (498/1917) of participants screened as Zone II on the AUDIT, 20.41% (402/1917) screened as Zone III, and the remaining 54% of participants screened as Zone IV (1029/1917). Participants also spent, on average, 41 min and 45 seconds per visit to the HSM site and visited, on average, 6 pages.

### Audit Score and Time in Program

To test hypothesis 1, a set of repeated measures ANOVAs was conducted to investigate any reductions in AUDIT score from program commencement to 1- and 3-month follow-up. Three separate ANOVAs were conducted to determine reduction in AUDIT score for participants who screened Zones II, III, or IV, respectively, at program commencement. Intention to treat data is reported, in that participants who failed to complete a follow-up survey at 1- or 3-month follow-up had their sign-up AUDIT score imputed at the follow-up points. Across all participants at program commencement, mean AUDIT score was 20.32 (SD 6.66), placing them on average in Zone IV of the AUDIT. At 1-month follow-up, the mean score was 12.60 (SD 9.95), and at 3-month follow-up, it was 8.90 (SD 8.86).

The assumption of sphericity was violated for all 3 ANOVAs; therefore, Greenhouse-Geisser *F* values and degrees of freedom are reported. For participants who screened Zone II at program commencement, there was a significant main effect of time, *F*_1.98,916.53_=401.28, *P*<.001, η_p_^2^=.45, with pairwise comparisons indicating AUDIT scores reduced significantly from program commencement to 1-month follow-up and then reduced significantly again at 3-month follow-up (see Table 1 for descriptive statistics). The result showed that participants who screened Zone II at program commencement had reduced to Zone I (low-risk drinking) at 3-month follow-up.

For participants who screened Zone III at program commencement, there was a significant main effect of time, *F*_1.92,771.14_=437.16, *P*<.001, η_p_^2^=.52, with pairwise comparisons indicating AUDIT scores reduced significantly from program commencement to 1-month follow-up and then reduced significantly again at 3-month follow-up (see Table 1 for descriptive statistics). The result showed that participants who screened Zone III at program commencement had reduced to borderline Zone I (low-risk drinking) at 3-month follow-up (M=7.67).

For participants who screened Zone IV at program commencement, there was a significant main effect of time, *F*_1.92,1975.28_=1067.23, *P*<.001, η_p_^2^=.51, with pairwise comparisons indicating AUDIT scores reduced significantly from program commencement to 1-month follow-up and then reduced significantly again at 3-month follow-up (see Table 1 for descriptive statistics). The result showed that participants who screened Zone IV (high-risk or dependent drinking) at program commencement had reduced to Zone II (risky or hazardous drinking) at 3-month follow-up. Overall, 58.3% of the sample had a reduction at 1-month follow-up and 89.3% had a reduction at 3-month follow-up.

**Table 1:** Alcohol Use Disorders Identification Test (AUDIT) score change across time by pre-AUDIT screening level.

Consumption	Pre, mean (SD)	1 month, mean (SD)	3 months, mean (SD)
Zone II^a^ (n=485)	11.92 (2.25)	7.59 (5.70)	5.39 (5.32)>
Zone III^b^ (n=402)	17.52 (1.08)	10.38 (7.43)	7.67 (6.99)
Zone IV^c^ (n=1029)	25.38 (4.20)	15.83 (11.11)	11.04 (10.14)

^a^Zone II: risky or hazardous consumption.

^b^Zone III: harmful consumption.

^c^Zone IV: high-risk or dependent consumption.

**Table 2:** Pearson correlations between Alcohol Use Disorders Identification Test (AUDIT) scores and engagement variables.

Variables	1-month AUDIT^a^ score change	3-month AUDIT score change	Sign in count	# of posts	Likes received	Comments received	Comments given	Following	Followed	Check-ins
1-month AUDIT score change	—	−.66^d^	−.12^d^	−.10^c^	−.13^d^	−.05	−.03	−.14^d^	−.11^c^	−.22^d^
3-month AUDIT score change	—	—	−.09^d^	−.07^b^	−.12^d^	−.04	−.03	−.09^b^	−.09^b^	−.23^d^
Sign-in count	—	—	—	.60^d^	.57^d^	.68^d^	.67^d^	.43^d^	.66^d^	.29^d^
# of posts	—	—	—	—	.84^d^	.83^d^	.73^d^	.40^d^	.76^d^	.27^d^
Likes received	—	—	—	—	—	.84^d^	.72^d^	.54^d^	.81^d^	.23^d^
Comments received	—	—	—	—	—	—	.93^d^	.44^d^	.81^d^	.16^d^
Comments given	—	—	—	—	—	—	—	.37^d^	.78^d^	.12^c^
Following	—	—	—	—	—	—	—	—	.75^d^	.14^d^
Followed	—	—	—	—	—	—	—	—	—	.26^d^
Check-ins	—	—	—	—	—	—	—	—	—	—

^a^AUDIT: Alcohol Use Disorder Identification Test.

^b^*P*<.05.

^c^*P*<.01.

^d^*P*<.001.

### Audit Score and Program Engagement

To test hypothesis 2, separate bivariate Pearson correlations were conducted to determine associations with 1- and 3-month follow-up AUDIT change scores, with various program engagement metrics. It was hypothesized that an increase in program engagement via following, sign-ins, posts, and comments would result in greater 1- and 3-month change scores.

Results indicate that, as expected, 1- and 3-month AUDIT change scores were negatively associated with more sign-ins, posts, received likes, following, followers, and check-ins (see Table 2), indicating the greater the reduction in AUDIT scores, the more of each of these engagement metrics participants had. The number of received comments or comments given did not correlate with 1- or 3-month AUDIT change.

### AUD Identification Test Score and Mood

To test hypothesis 3, that 1-month and 3-month AUDIT change would be negatively correlated with mood (ie, worse mood would result in higher AUDIT scores), and positively correlated with slip-ups (ie, more slip-ups would result in higher AUDIT scores), separate bivariate Pearson correlations were conducted. To run the analysis, the good and bad check rate was calculated, with good check indicating the proportion of check-ins where the participant indicated they had not had a drink that week, and bad check indicating the proportion of check-ins where the participant indicated they did have a drink that week.

**Table 3:** Pearson correlations between Alcohol Use Disorders Identification Test (AUDIT) scores, mood, and check-in ratings.

Variables	1-month AUDIT^a^ score change	3-month AUDIT score change	Good check	Bad check	Positive check	Negative check
1-month AUDIT score change	—	.66^d^	.09^c^	−.25^d^	−.23^d^	−.02
3-month AUDIT score change	—	—	.07^b^	−.26^d^	−.24^d^	−.01
Good check	—	—	—	−.01	.08^b^	.68^d^
Bad check	—	—	—	—	.96^d^	.21^d^
Positive check	—	—	—	—	—	.17^d^
Negative check	—	—	—	—	—	—

^a^AUDIT: Alcohol Use Disorder Identification Test.

^b^*P*<.05.

^c^*P*<.01.

^d^*P*<.001.

Moreover, positive and negative check-in was calculated, with positive check-in representing the proportion of check-ins where the participant indicated they had a positive mood that week, and negative check-in representing the proportion of check-ins where the participant indicated they had a negative mood that week.

Results indicated that the good check rate was positively correlated with 1- and 3-month AUDIT change scores, and bad check was negatively correlated with 1- and 3-month AUDIT change scores (see Table 3). Contrary to expectations, this indicated that more slip-ups were correlated with lower 1-and 3-month AUDIT change scores, and less slip-ups were associated with higher 1- and 3-month AUDIT change scores. This indicated those who had reduced their AUDIT scores had more slip-ups during the program, than those whose AUDIT scores had increased. However, as expected, positive check-in was negatively correlated with AUDIT change scores, indicating that more check-ins where mood for the week was positive were associated with greater reduction in AUDIT scores. Interestingly, negative check-in (ie, the proportion of check-ins where mood that week was low) was not correlated with either 1- or 3-month AUDIT change scores.

## Discussion

### Principal Findings

Alcohol use is a major social and public health issue in Australia and around the world, with the major barriers for accessing treatment including accessibility of services, stigmatization, low motivation, and cost of treatment [7]. Alarmingly, there is a significant gap between those who exhibit problematic alcohol use and those that receive some form of treatment [6]. Furthermore, those with less severe alcohol abuse are not offered any form of intervention [8]. It is clear that emerging technologies may offer a low-cost, accessible alternative that may be able to support this *hidden* population; however, limited research exists in regard to the efficacy of blogging and social media engagement on alcohol consumption.

This study aimed to explore whether participation in the HSM platform is associated with changes in alcohol consumption. The HSM program asks participants to set a goal to stop or reduce their drinking for a set time, and to record their reflections and progress through blogs, while being connected with others within the community working toward similar goals.

As with previous research on the platform, participants were predominately female, and were beginning the program with predominately harmful or high-risk consumption levels [36]. Results revealed that individuals who reported hazardous and harmful consumption levels and who engaged in the HSM program reported a significant decrease in alcohol consumption, moving to low-risk consumption levels 4 months following program commencement. Those who reported high-risk or dependent consumption levels before program engagement experienced the biggest reduction, moving to risky consumption 4 months following program commencement. These reductions in risk were maintained by participants in each AUDIT screening zone, 7 months following program commencement. Given the cost and wait times for traditional treatment programs, this is a significant finding, suggesting long-term behavior change can occur through the use of an easily accessible Web-based program. Furthermore, it is important to note that this program was more effective for those drinking to high-risk and hazardous levels.

As expected, those who engaged in the program more had significantly lower levels of alcohol consumption at 1- and 3-month follow up. In particular, significant improvements were seen for those who signed-in more, posted more blogs, received more “likes” from other community members, followed more community members, had more followers, and completed more check-ins.

It appears the engagement with the community and peer support was a key ingredient in the successful behavior change of HSM participants, with such peer-to-peer communities being described as one of the most transformational features of the internet [46]. Through this model, individuals with multiple barriers have the opportunity to connect and create supportive communities, with narrative expression having demonstrated psychological benefits for people experiencing chronic illness—allowing reflection, connection, and meaning-making [47-49].

The benefits of such expressive writing may include acknowledging and validating experiences, communicating the experience of recovery and relapse, and allowing for reflection of the current reality. The reflective nature of narrating personal experiences encourages self-disclosure and sharing of thoughts and emotions, as well as encouraging the participant to disclose facts about themselves and reduce inhibition [49], as well as providing an opportunity to externalize their inner experiences [50]. When this content is shared within a trusted and supportive community, it appears that participants feel validated, and in turn offer support and validation to other members of the community, thus creating a network of participants working toward the same goal of long-term behavior change [46,51,52].

The last hypothesis was that 1- and 3-month AUDIT scores would be negatively correlated with mood and positively correlated with slip-ups. However, results showed that negative mood was not related to alcohol consumption, but positive mood was significantly related to lower alcohol consumption. This is an interesting finding and warrants further investigation, particularly around what specific mood states predict drinking to excess, rather than simply positive versus negative emotions. Finally, results also show that more slip-ups were associated with lower alcohol consumption. This suggests that slip-ups provided more opportunities to learn about triggers, responses, and strategies to persist with rather than just focusing solely on staying sober. When supporting individuals experiencing a slip-up or lapse, it is therefore important to take advantage of the learning opportunity and facilitate processing, reframing, and learning rather than focusing on or trying to resolve feelings of weakness, guilt, and hopelessness.

### Limitations

Despite the findings in this study, a number of limitations should be recognized. As noted, this study did not include a control group or benchmark group to compare to, and did not control for variables such as additional treatment (psychological or pharmacological) and support. Although this limits the conclusions that can be drawn from the analyses, it has provided preliminary data to support the currently underway research projects utilizing best practice research designs. Additionally, although results showed participants were spending an average of 41 min per visit on the HSM site, the exact nature of how they spent their time on the site is unknown. They may have been predominately reading blog posts, writing their own blog posts, or commenting on other members’ posts. Future research exploring the different functions of the program must address this limitation.

Finally, mood was measured by a single visual scale simply describing positive and negative mood through the use of a happy or sad face dichotomy. It is recommended that a more descriptive and validated measure is used in the future, to allow more reliable evaluation of mood and mood states. Although more comprehensive measures are recommended for assessment of mood, it can also be argued that traditional questionnaires assessing mood can present issues such as cognitive load, overall burden, and longer completion times [53,54]. Previous well-validated pictorial measures of mood include the Self-Assessment Manikin, a set of 3 pictorial assessment scales measuring pleasure, arousal, and dominance [55], as well as the Smileyometer [56], the AffectButton [57], and EmoCards [58]. Each of these measures aims for respondents to report their affective state quickly and accurately. An additional benefit to these scales is that they can be used reliably across cultures [59], as well as being time-efficient ways for respondents to convey their affective state.

Future measures may include both multidimensional tools to assess mood state, as well as brief, unidimensional pictorial measures. As mood disorders have a long-established link to alcohol use [60-62], it will be useful in future research to also utilize measures such as the Depression Anxiety and Stress Scale and the K10, which are more able to assess the multidimensional nature of mood disorders and co-occurring depression, anxiety, and stress.

### Conclusions

The findings of this study highlight a number of future directions for program development and future investigation into the elements involved in the HSM program. These findings suggest that it is possible to bring about significant change in alcohol consumption through the use of an online platform and Web-based community. The large number of participants drinking to high-risk or dependency levels has, however, highlighted the need for further accessible targeted clinical support.

The results of this study indicate that being part of a supportive Web-based community, as well as having the opportunity to reflect on past experiences, may provide participants with the resources needed to create lasting behavior change. This is a crucial service for the *hidden* population of individuals with problematic alcohol use who do not seek out traditional services, or are not identified as being in-need of tailored support based on assessments or lack of, by their primary health providers. An added benefit to this is that the Web-based community is highly accessible, in that members are able to use it from their mobile phones or laptops, as well as utilizing the well-validated model of a supportive “sober” network. This can be compared with the traditional model of alcohol treatment, which is quite rigid in its service delivery and approaches—a shortcoming that is reflected in its poor engagement and outcomes [63]. It is hoped that with the introduction of new technologies, the social support model will continue to be utilized and the *hidden* population of high-risk drinkers will be able to access ongoing and effective support.

## Abbreviations

ANOVA: analysis of variance
AUD: alcohol use disorder
AUDIT: Alcohol Use Disorders Identification Test
HSM: Hello Sunday Morning
SBI: screening and brief intervention
