# Metadata Correction: Alcohol Consumption Reduction Among a Web-Based Supportive Community Using the Hello Sunday Morning Blog Platform: Observational Study

**DOI:** 10.2196/11288

**Published:** 2018-09-10

**Authors:** Jessica Jane Louise Kirkman, Briony Leo, Jamie Christopher Moore

**Affiliations:** 1 Hello Sunday Morning Surry Hills Australia

The authors of the paper “Alcohol Consumption Reduction Among a Web-Based Supportive Community Using the Hello Sunday Morning Blog Platform: Observational Study” (J Med Internet Res 2018;20(5):e196) wish to amend the contact number listed on the paper. In the “Corresponding Author” section, the telephone number was listed as 61 422924764, but this number has now been changed to 61 1300 403 196.

The correction will appear in the online version of the paper on the JMIR website on September 10, 2018, together with the publication of this correction notice. Because this was made after submission to PubMed, PubMed Central, and other full-text repositories, the corrected article also has been resubmitted to those repositories.

